# Therapeutic Hypothermia Protects Against Heat Stroke-Induced Arterial Hypotension via Promoting Left Ventricular Performance in Rats

**DOI:** 10.7150/ijms.39745

**Published:** 2020-02-10

**Authors:** Wen-Ching Ko, Cheng-Hsien Lin, Jie-Jen Lee, Ching-Ping Chang, Chien-Ming Chao

**Affiliations:** 1 Department of Surgery, Mackay Memorial Hospital, Taipei, Taiwan; 2 Department of Medicine, Mackay Medical College, New Taipei, Taiwan; 3 Department of Medical Research, Chi Mei Medical Center, Tainan, Taiwan; 4 Department of Intensive Care Medicine, Chi Mei Medical Center, Liouying, Tainan 73657, Taiwan; 5 Department of Nursing, Min-Hwei College of Health Care Management, Tainan, Taiwan

**Keywords:** myocardial injury, therapeutic hypothermia, heatstroke, cardiac mechanical efficiency, arterial elastance

## Abstract

We aimed to ascertain whether therapeutic hypothermia (TH) acts as cardioprotective management for heat stroke (HS). Adult male rats under general anesthesia were exposed to whole-body heating (43°C for 70 min) to induce HS. Rats with HS displayed hyperthermia (core body temperature 42°C vs. 36°C); hypotension (30 mmHg vs. 90 mmHg mean arterial blood pressure); suppressed left ventricular (LV) performance (stroke volume 52 μl/min vs. 125 μl/min), ejection fraction (0.29% vs. 0.69%), relaxation factor (72 ms vs. 12 ms), and arterial elastance (0.31 mmHg/ μl vs. 10 mmHg/ μl); increased myocardial injury markers (e.g., creatine kinase-MB: 86 U/L vs. 24 U/L, cardiac troponin I: 3.08 ng/ml vs. 0.57 ng/ml); increased myocardial oxidative stress markers (e.g., malondialdehyde: 6.52 nmol/mg vs. 1.06 nmol/mg, thiobarbituric acid-reactive substances: 29 nmol/g vs. 2 nmol/g); decreased myocardial antioxidants (e.g., superoxide dismutase: 6 unit/mg vs. 17 unit/mg, reduced glutathione: 0.64 nmol/mg vs. 2.53 nmol/mg); increased myocardial proinflammatory cytokines (e.g., tumor necrosis factor-α 3200 pg/ml vs. 1000 pg/ml, interleukin-6: 668 pg/ml vs. 102 pg/ml); and increased cardiac damage scores (2.2 vs. 0.3). TH therapy significantly reversed the following conditions: HS-induced hyperthermia (37.5°C core body temperature), hypotension (71 mmHg), suppressed LV performance (stroke volume: 97 μl/min, ejection fraction: 0.65%, relaxation factor: 39 ms, and arterial elastance: 0.99 mmHg/μl), increased myocardial injury markers (e.g., creatine kinase-MB: 37 U/L, cardiac troponin I: 1.06 ng/ml), increased myocardial oxidative stress markers (e.g., malondialdehyde: 2.68 nmol/mg, thiobarbituric acid-reactive substances: 12.3 nmol/g), decreased myocardial antioxidants (e.g., superoxide dismutase: 13.3 unit/mg, reduced glutathione: 2.71 mmol/mg), increased myocardial proinflammatory cytokines (e.g., tumor necrosis factor-α 1500 pg/ml, interleukin-6: 108 ng/ml); and increased cardiac damage scores (0.9). We thus conclude that TH protects against HS-induced arterial hypotension by promoting LV performance in rats. These results add to the literature regarding the use of TH as cardioprotective management for HS.

## Introduction

Temperature elevation (either spontaneous/ infection-related or induced by external warming) can exacerbate all types of neurologic injury (cardiac arrest, ischemic stroke, traumatic brain injury, and hemorrhagic stroke) 1. Therapeutic hypothermia (TH) is an effective therapy for comatose patients, improving cardiac and neurologic outcomes after the return of spontaneous circulation following cardiac arrest 2-8. After the return of spontaneous circulation following cardiac arrest, all comatose adult patients should receive target temperature management with a target temperature between 32°C and 36°C 9. Patients with heat stroke (HS) present electrocardiographic abnormalities, including a predominant “S” wave and a depressed “ST” segment 10. HS rats also display subcellular alterations (e.g., mitochondrial hypertrophy and intracellular edema) in their myocardial tissue 11. *In vitro* studies have also demonstrated that heat stress causes time- and dose-dependent apoptosis, necrosis, and autophagy in rat cardiomyocytes 12. TH is the current therapy of choice for HS because no pharmacologic agent is currently available 13-15. Although the true mechanisms underlying the beneficial effect of TH on HS reactions remain unclear, evidence has accumulated to indicate that TH may improve outcomes of HS via reducing cardiac dysfunction 16, 17. Indeed, our previous findings showed that after the onset of HS, animals displayed hypotension and altered cardiac protein profiles, which could be reversed by TH. This raises the possibility that cardiac dysfunction contributes to HS genesis, which can be ameliorated by TH 18.

In this study, to address this question, we used a rat model of HS 19 that exhibits hyperthermia, arterial hypotension, suppressed left ventricular (LV) performance and cardiac inflammatory and oxidative damage. We tested the hypothesis that TH protects against HS-induced arterial hypotension via promoting LV performance by pressure-volume loop analyses in HS rats. Based on the instructions provided by previous studies, our HS animals received target temperature management (32-36°C).

## Materials and Methods

### Ethics Statement

This study strictly adhered to the recommendations in the Guide for the Care and Use of Laboratory Animals of the Ministry of Science and Technology (MOST) of the Republic of China. The protocol was approved by the Chi Mei Medical Center Institutional Review Board for Animal Care and Use (Assurance Number: 106121110). All effects were made to minimize the suffering of the experimental rats.

### Animals and Surgery

Sixty adult male Sprague-Dawley rats (250±12 g) obtained from BioLASCO Taiwan Co. and housed at an ambient temperature of 22±1°C with a 12-h light-dark cycle. Pellet rat chow and tap water were available *ad libitum*. Rats were anesthetized with an intraperitoneal (i.p.) dose of pentobarbital sodium (60 mg/kg). The femoral artery of anesthetized rats was cannulated with polyethylene tubing (PE-60) for measurement of mean arterial blood pressure (MABP) and HR. Both MABP and HR were monitored continuously using a pressure transducer (ADInstruments Inc., Springs, CO, USA) and a chart recorder (ADInstruments Inc.).

### HS Induction

HS was induced by exposing anesthetized rats in a folded heating pad at 43°C controlled by 43°C water-circulating pad for 70 min as detailed previously 20. Then, the heated rats were allowed to recover at room temperature of 26°C. Twenty minutes following the termination of the 70-min heat stress, all the heated rats displayed both excessive hyperthermia (~42.0°C) and arterial hypotension (~30mm Hg), indicating the occurrence of HS 21 (please see Figure 2).

### TH

Before the start of the thermal experiments, the body core temperature of all anesthetized animals was maintained at 36.2°C using a water-circulating folded heating pad. Immediately after the termination of heat stress, all heated rats were subjected to TH (16°C water-circulating folded cooling pad for 30 min). Their body core temperatures were decreased to ~38°C and then maintained at 36°C by a folded heating pad at 26°C in a room at 26°C.

### Experimental Groups and Experimental Procedures

The animals were assigned to one of the following 3 groups: the NC group (n=10): rats with body core temperature maintained at 36°C in a room at 26°C throughout the experiment; the HS+non-TH group (n=10): rats treated with HS (43°C for 70 min) followed by non-TH (26°C for 30 min); and the HS+TH group (n=10): rats treated with HS (43°C for 70 min) followed by TH (16°C for 30 min). The survival rate was obtained for each group. All 3 groups of rats were subjected to pressure-volume (P-V) loop analyses.

### P-V Loop Analyses

A 1.2 F catheter-tip pressure transducer (Scisense, London, Ontario, Canada) was inserted into the right carotid artery to measure the baseline arterial pressure and then fed retrograde into the left ventricle to record baseline hemodynamics in the closed chest with the ADVantage^TM^ (Scisense, London, Ontario, Canada). The signals of P and V were visually checked for quality and recorded for later analysis 22, 23. The following hemodynamic parameters were calculated: HR, heart rate (beats/min); ESP, LV end-systolic pressure (mmHg); EDP, LV end-diastolic pressure (mmHg); SV, stroke volume (μl); ESV, LV end-systolic volume (μl); EDV, LV end-diastolic volume (μl); CO, cardiac output (μl/min); EF, ejection fraction (%); maximal dp/dt, (mmHg/s); Pmax, maximum LV pressure (mmHg); Vmax, maximum LV volume (μl/s); SW, stroke work (mmHg/ml); minimum dp/dt (mm Hg/s); Pmin, minimum LV pressure (mmHg); Tau (γ) Glantz time constant of ventricular relaxation (ms).

### H&E Staining

At twenty minutes after the onset of HS, both the blood and heart tissue were collected from all groups of rats under anesthesia. The blood samples were centrifuged to obtain the serum for further biochemical assays. The heart tissue was immersed in 10% neutral formalin, dehydrated through graded alcohols and embedded in paraffin wax. Ten-micrometer thick sections were cut, stained with H&E and examined under a light microscope (Carl Zeiss GmbH, Göttingen, Germany). Myocardial damage was scored using published morphologic criteria 24: 0, no damage; 1 (mild), interstitial edema and localized necrosis; 2 (moderate), widespread myocardial cell swelling and necrosis; 3 (severe), necrosis with contraction bands and compressed capillaries; or 4 (highly severe), diffuse necrosis with contraction bands, compressed capillaries, and hemorrhage. Histological sections were evaluated in a blinded manner by two examiners. The total cardiac damage score for each heart was calculated as an average of all the component injury scores.

### Estimation of Myocardial Injury Markers

Myocardial injury was assessed in rats after 70 min of heat stress plus 20 min of room temperature recovery via estimating specific biomarkers, cardiac troponin I, lactate dehydrogenase, and creatine kinase-MB isoenzyme in serum samples using commercially available standard kits. All measurements were performed according to the manufacturer's instructions.

### Estimation of Oxidative Stress and Antioxidant Defense System

The supernatant obtained from the 10% homogenate of heart tissue was used for the estimation of the end product of lipid peroxidation (malondialdehyde, MDA) reacted with thiobarbituric acid in acidic conditions and formed a pink colored chromophore measured at 532 nm using a microplate reader 25. Thiobarbituric acid-reactive substances (TBARS) were expressed as nM/g tissue. Superoxide dismutase (SOD) activity in the heart tissue was measured using the SOD assay kit 26. We used the method of Sinha 27 to measure the activity of catalase in the heart tissue. Additionally, 5,5'-dithiobis (2-nitrobenzoic acid) (DTNB) was used to estimate the levels of reduced glutathione (GSH) in the heart tissue 28.

### Estimation of Two Pro-inflammatory Cytokines (TNF-α and IL-6) and One Anti-inflammatory Cytokine (IL-10)

We estimated the levels of two pro-inflammatory cytokines (TNF-α and IL-6) and one anti-inflammatory cytokine (IL-10) in the heart tissue homogenate of control and experimental groups by specific ELISA kits, according to the manufacturers' instructions.

### Statistical Analysis

The results for multiple independent experiments are expressed as the mean±standard deviation (SD). Survival rates were compared using Kaplan-Meier analysis followed by the log-rank test. One-way analysis of variance followed by the Student-Newman-Keuls post hoc test was performed to analyze differences between multiple groups. *P*<0.05 was considered significant.

## Results

### Prolonging the Survival Rate and Reducing Excessive Hyperthermia and Arterial Hypotension in HS Rats by TH

**Figure 1:**
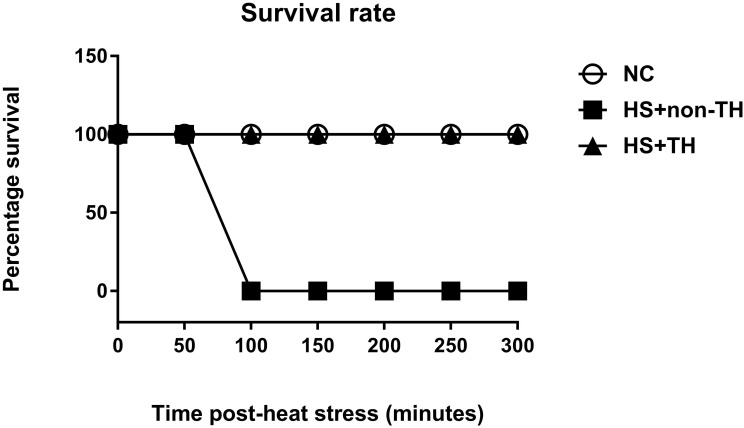
Kaplan-Meier analysis followed by log-rank tests were performed to determine the percent survival in NC rats (⚪), in heated rats without TH following HS (HS+non-TH) (◼) and in heated rats with TH (HS+TH) (▲). Data are expressed as the means±SD of 10 rats per group. *P<0.001, HS+non-TH vs. NC; ^+^P<0.001, HS+TH vs HS+non-TH.

**Figure 2:**
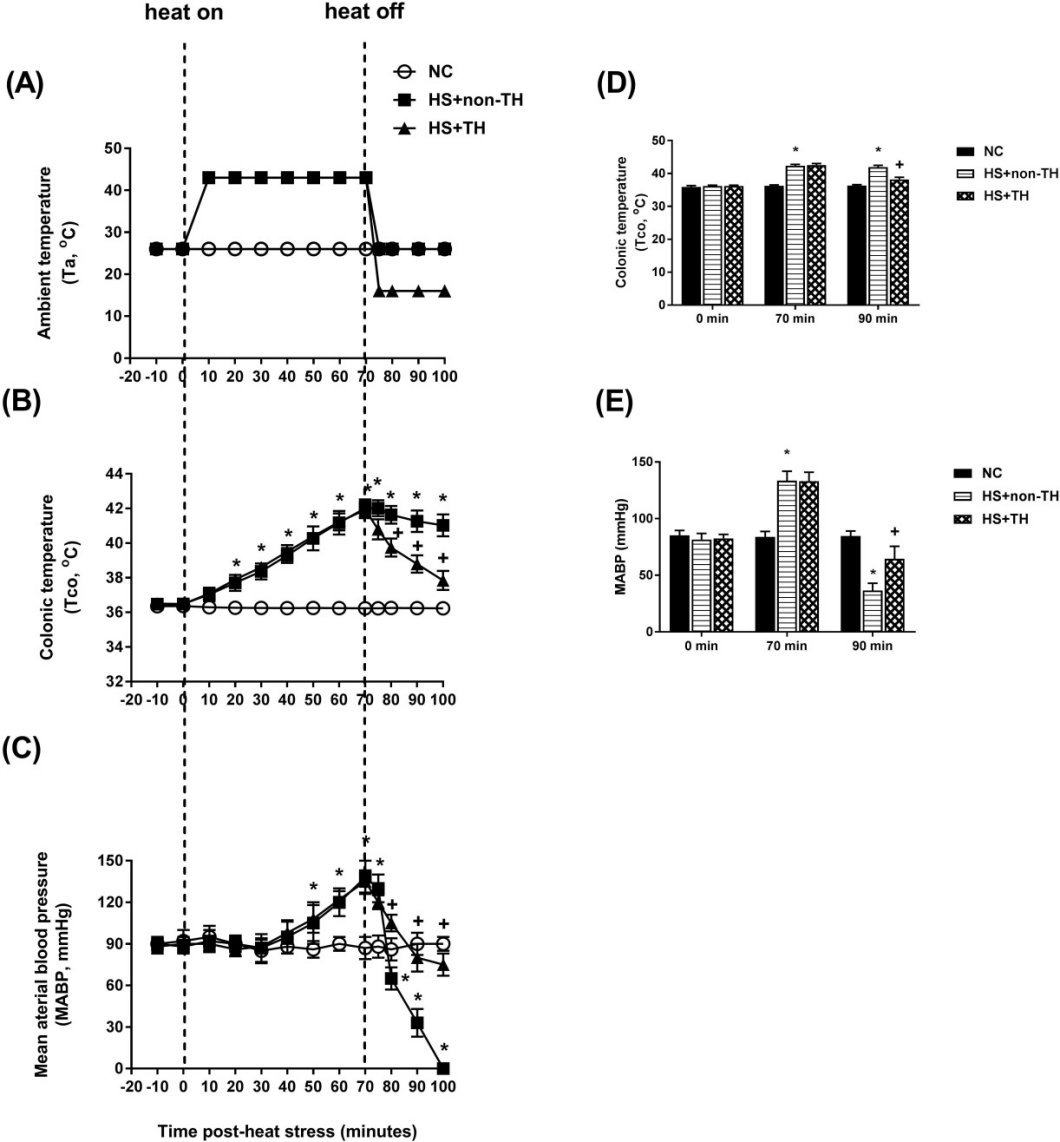
Time course of the change in ambient temperature (A, Ta), colonic temperature (B, Tco) and mean arterial blood pressure (C, MABP) in NC rats (⚪), in rats without TH following HS (HS+non-TH) (◼), and in rats receiving TH following HS (HS+TH) (▲). The Tco (D) and MABP (E) were obtained 0, 70, and 90 min after the initiation of heat exposure (ambient temperature in non-heated controls ) in heat stroke rats. All heated groups were exposed to heat (43 ^o^C) for exactly 70 min and were then allowed to recover at room temperature (26 ^o^C). Data are expressed as the means±SD of 10 rats per group. *P<0.01, HS+non-TH vs. NC; ^+^P<0.05, HS+TH vs. HS+non-TH.

The potential reduction in the lethality of HS in rats was first analyzed by determining their survival rates. Heat stress (43°C for 70 min) significantly reduced the percent survival from 100% in the normothermic control (NC) group rats to 0% in the HS rats without TH (HS+non-TH) (P<0.001; Figure 1). In contrast, the HS+TH group rats exhibited a significant (P<0.001; Figure 2) increase in the percent survival (100%). In addition, the occurrence of excessive hyperthermia (~42°C vs. ~38°C colonic temperature), as well as arterial hypotension (~30 mmHg vs. 60 mmHg) at 20 min following the termination of HS (or 90 min after heat-stroke onset), was significantly reduced by TH, as demonstrated in the HS+TH group rats (P<0.01; Figure 2).

### TH-induced Reversion of Suppressed LV Performance in HS Rats

**Figure 3:**
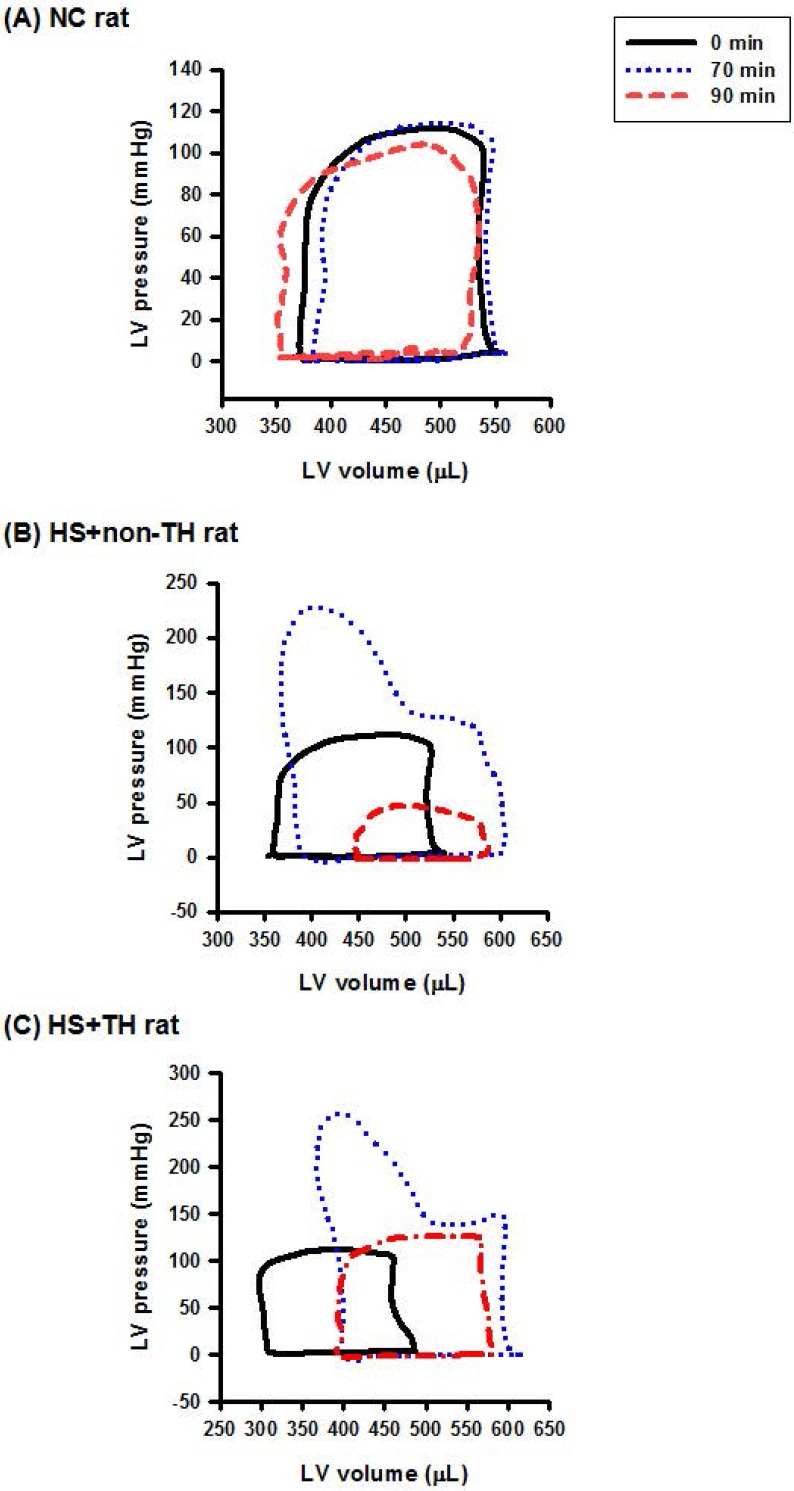
Representative LV pressure-volume loops obtained from the steady-state of (A) an NC rat, (B) an HS+non-TH rat, and (C) an HS+TH rat at time “0 min” (―), time “70 min” (…), and time “90 min” (**-·-·**) after onset of heat stress. As depicted in (B), the HS+non-TH rat displayed a characteristic right shift and declined in the amplitude of the pressure-volume signal in the pressure-volume loops at the time “90 min”. The abnormal amplitude of the pressure-volume signal in the pressure-volume loop at the time “90 min” (or the time point for the onset of HS) was significantly reversed by the TH, as demonstrated in HS+TH rats. The definitions of the group abbreviations are provided in the legend of Figure 1.

As shown in both Figure 3 and Table 1, compared with the NC group rats, the HS+non-TH groups had significantly lower values of heart rate (HR) (53±4 beats/min vs. 349±5 beats/min), end-systolic pressure (ESP) (15±1 mmHg vs. 119±3 mmHg), CO (2982±22 μl/min vs. 44594±37 μl/min), EF (0.29±0.02% vs. 0.69±0.04%) and arterial elastance (Ea) (0.31±0.02 mmHg/μl vs. 10.1±0.3 mmHg/μl) but had significantly higher values of relaxation factor (γ) (72±1 ms vs. 12±0 ms). Notably, the suppressed left ventricle hemodynamics in the HS+non-TH group rats were significantly reversed by TH therapy in the HS+TH group rats: HR (512 ±12 beats/min), ESP (71±4 beats/min), CO (45740±35 μl/min), EF (0.65±0.03%), Ea (0.99±0.02 mmHg/μl), and γ (39±1 ms) (Table 1 and Figure 3).

### TH Attenuates HS-induced Pathological Changes in the Myocardial Tissues

**Figure 4:**
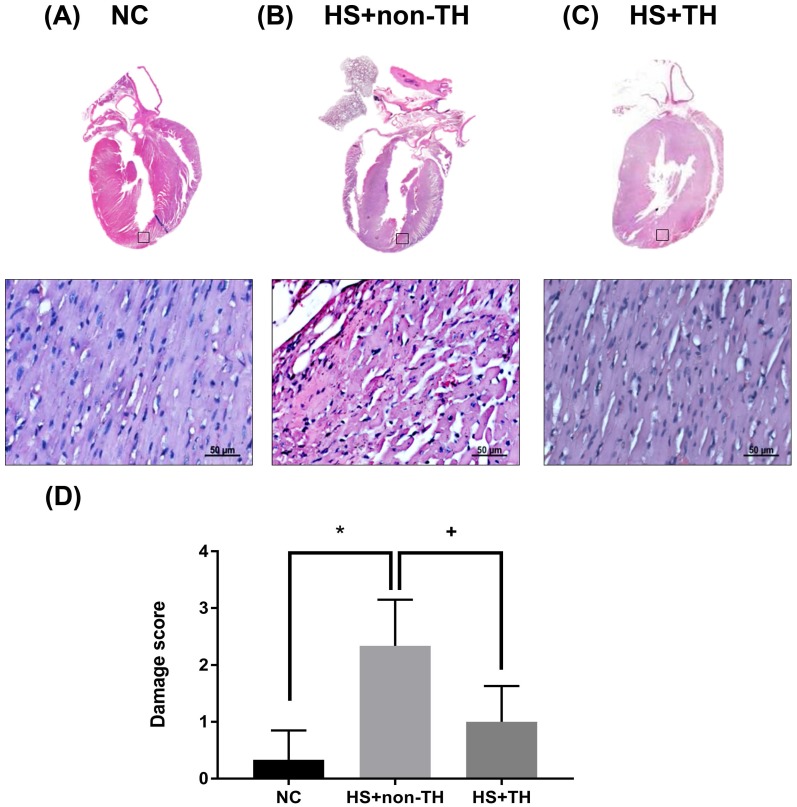
Histological analysis of myocardial tissues. Representative hematoxylin and eosin (H&E) staining results for myocardial sections in rats of the (A) NC, (B) HS+non-TH and (C) HS+TH groups. (D) Semiquantitative analysis of H&E staining in the rats (n=6 per group). *P<0.05, compared with NC, +P<0.05, compared with HS+non-TH. At twenty min after HS onset, gross observation, and 400x magnification with H&E staining revealed apparent differences in the degree of injury between the different experimental groups. Scale bars: 50 µm.

The cardiac histology of the NC control rats revealed a normal appearance showing normal and centrally arranged nuclei, the connective tissue also appeared normal, and the cardiac muscle fibers were well arranged. When compared with the NC group (Figure 4 A), the HS+non-TH group of rats revealed significant pathological changes in the myocardial tissues (Figure 4 B). The changes observed in the HS+non-TH group included cellular edema or atrophy, inflammatory cell infiltration, coagulative necrosis, and liquefactive necrosis (Figure 4 B). In contrast, the myocardial tissue from the HS+TH group exhibited less cellular edema, mild leukocytic infiltration, and muscle necrosis (Figure 4 C). Semi-quantitative assessment of the histological lesions also confirmed that the HS+TH group had a significantly higher cardiac damage score than did the HS+non-TH group (Figure 4 D).

### Reversing the Increased Concentrations of Myocardial Injury Markers in HS Rats by TH Therapy

Compared with the NC group rats, the HS+non-TH group rats had significantly increased serum levels of creatine kinase-MB (86±9 U/L vs. 24±4 U/L), lactate dehydrogenase (155±12 U/L vs. 51±8 U/L), and cardiac troponin I (3.08±0.72 ng/ml vs. 0.57±0.08 ng/ml) (Table 2). However, compared with the HS+non-TH group rats, the HS+TH group rats had significantly decreased serum levels of creatine kinase-MB (37±6 U/L), lactate dehydrogenase (76±9 U/L) and cardiac troponin I (1.06±0.11 ng/ml) (P<0.05; Table 2).

### Reversing the Increased Levels of Cardiac Oxidative Stress in HS Rats by TH

Compared with the NC group rats, rats in the HS+non-TH group had significantly increased cardiac levels of MDA (6.52±1.02 nmol/mg vs. 1.06±0.19 nmol/mg) and TBARS (29±4 nmol/g vs. 2±1 nmol/g) (Table 2; P<0.05). In contrast, compared with the NC group rats, the HS+non-TH group rats had significantly decreased cardiac levels of SOD (6±2 units/mg vs. 17±3 units/mg), catalase (5±2 nM H_2_O_2_ consumption/min/mg protein vs. 9±2 nM H_2_O_2_ consumption/min/mg protein) and GSH (0.64±0.32 mmol/mg protein vs. 2.53±0.24 mmol/mg protein) (Table 2, P<0.05). TH significantly reversed both the increased cardiac levels of both MDA (2.68±0.63 nmol/ng) and TBAS (12±3 nmol/g) and the decreased cardiac levels of SOD (13±3 unit/mg), catalase (10±3 nM), and GSH (2.71±0.26 mmol/mg) (Table 2).

### Reversing the Increased Cardiac Levels of Inflammatory Status in Heatstroke Rats by TH

Compared with the NC group rats, the HS+non-TH group rats had a significant increase in the cardiac levels of both TNF-α (3200±310 pg/ml vs. 1000±380 pg/ml) and IL-6 (668±85 pg/ml vs. 102±24 pg/ml) as well as a significant decrease in the cardiac levels of the anti-inflammatory cytokine IL-10 (992±227 pg/ml vs. 3008±660 pg/ml) (Table 2). However, TH significantly reversed these alterations induced by HS (TNF-α, 1500±320 pg/ml vs. 3200±310 pg/ml; IL-6, 108±35 pg/ml vs. 668±85 pg/ml; IL-10, 2955±441 pg/ml vs. 992±227 pg/ml) (Table 2).

## Discussion

In the present study, we subjected anesthetized rats to severe heat stress (43°C for 70 min) to induce HS 19. The occurrence of body core temperature elevation (above 42°C) and decreased mean arterial pressure (below 35 mmHg) was taken as the time point of HS onset. At this time point, our heated rats displayed excessive hyperthermia, arterial hypotension, decreased stroke volume, decreased cardiac output, decreased ejection fraction, decreased stroke work, increased relaxation factor, and decreased arterial elastance. Additionally, HS rats had an increase in the serum levels of several cardiac damage markers, including cardiac troponin I, lactate dehydrogenase, and creatinine kinase-MB. The heated rats also had increased cardiac levels of pro-inflammatory cytokines such as TNF-α and IL-6 and decreased cardiac levels of the anti-inflammatory cytokine IL-10. Furthermore, HS rats displayed increased cardiac extents of lipid peroxide formation (revealed by increased cardiac levels of both MDA and TBARs 29 and decreased cardiac levels of SOD, catalase, and GSH (reflecting the decreased capability of antioxidant enzymes) 30-32. Notably, decreasing the body core temperature from ~42.5°C to ~38°C by TH significantly reversed the hypotension and reduced the myocardial inflammatory and oxidative injury in HS rats.

TH (32 ^o^C) reduced the inflammatory response following ischemia/reperfusion injury in rat hearts 33. TH decreased the inflammatory cytokines in the risk zone of the heart, which included the IL-6 and TNF-α, inducible nitric oxide synthase, and others. Additionally, TH reduced levels of many inflammatory mediators including radical oxygen species (ROS) and pro-inflammatory cytokines (such as IL-1β, IL-6, and TNF-α) 34. In our present study, we choose to measure two pro-inflammatory cytokines (IL-6 and TNF-α) as well as one anti-inflammatory cytokine (IL-10) for the elucidation of inflammatory status. At the same time, we choose to measure cardiac contents of MDA and TBARS for the elucidation of lipid peroxidation formation and cardiac contents of SOD, catalase, and GSH for the elucidation of the decreased capability of anti-oxidant enzymes.

As mentioned in the Introduction section, in patients who have been successfully resuscitated after cardiac arrest due to ventricular fibrillation, TH causes both a favorable neurologic outcome and reduced mortality 35. Epidemiological studies of HS have also recommended that the goal of clinical therapies be to normalize the body (core and skin) temperature and the function of multiple vital organs as rapidly as possible 13-15. Indeed, our present study confirmed that TH significantly attenuated myocardial injury, multiple organ damage, and lethality in heated rats. TH adopted immediately after HS onset significantly increased the percent survival from 0% to 100% in heated rats. Maintaining cardiac function by TH is vital for preserving adequate perfusion of vital organs and for decreasing multiple organ damage, especially brain damage, during HS. The goal of clinical therapy for heat stroke is to normalize body temperature, but approximately 30% of HS survivors experience disabilities as well as neurological dysfunction 13-15. Multiple organ dysfunction syndromes continue to manifest in HS patients after TH 13-15. Numerous preclinical studies of TH have suggested optimal cooling conditions, such as depth duration, and a temporal therapeutic window for effective protection 36. To administer such TH in HS, more investigation is still needed to understand the optional clinical setting better. For example, the induction of TH of 32-34 ^o^C at the onset of ischemia provides effective cardioprotection in male rodent models of acute myocardial infarction 37. In the present study, we choose TH of 32 ^o^C, without TH of a lower temperature for the treatments in male rats. This is a limitation of the present study. Additionally, this might be another limitation of the present study that we did not use female rats for the treatments.

Our present results are consistent with many previous findings. For example, in murine hemorrhagic shock, TH can modulate and release cardiac pro-inflammatory cytokines 38. In a rat model with controlled hemorrhagic shock, TH can better preserve systolic and diastolic functions 39 and can attenuate myocardial apoptosis 20, 40. Hypothermia treatment preserves mitochondrial integrity and viability of cardiomyocytes after ischemia-reperfusion injury 41. TH treatment can ameliorate cardiac dysfunction and help preserve both mitochondrial integrity and electron transport activity for postcardiac arrest myocardial dysfunction 41. Long noncoding RNA upregulated in hypothermia-treated cardiomyocytes protects against myocardial infarction by improving mitochondrial function 42. Although percutaneous coronary intervention (PCI) reduces infarct size, PCI-mediated tissue reperfusion of ischemic tissue causes irreversible myocardial damage 43. Therefore, new therapies for acute myocardial infarction should consider the reduction of both ischemia- and reperfusion-mediated tissue injuries 44, 45. During acute myocardial ischemia, pharmacological compounds are unable to reach the ischemic tissues due to limited blood flow. Combination therapy with TH could provide a new strategy for early intervention. Indeed, in this study, we demonstrated that TH is cardioprotective during myocardial ischemia (due to hypotension) right at the onset of HS.

The induction of mild hypothermia of 32-34 ^o^C at the onset of ischemia provides effective cardioprotection in an experimental model of acute myocardial infarction 46. In contrast, induction of cooling at reperfusion alone does not reduce infarct size both in the experimental 46 and clinical settings 47. In the present study, immediately after the termination of heat stress (or 70 min after the start of heat stress), all heated rats were subjected to TH (16 ^o^C water-circulating folded cooling pad for 30 min)(Figure 2). In heated rats, the values of MABP started to fall at 70 min and downed to a value of ~30 mmHg from the control levels of ~96 mmHg at 90 min. Therefore, TH was performed in the present study at a suitable time for the treatment but not to be late.

The injured brain stimulates innate immune molecule production and these induced molecules, including radical oxygen species (ROS), protease, and pro-inflammatory cytokines (such as IL-1β, IL-6, and TNF-α) can activate more inflammatory cells, leading to a vicious cycle of death and inflammatory activation 48. Indeed, TH lower numbers of infiltration neutrophils and activated macrophages in the ischemia and reduces levels of many inflammatory mediators including ROS 49, pro-inflammatory cytokines (such as IL-1β, IL-6, and TNF-α) 50 and others. In our present study, TH may offer the potential to interrupt the ischemic cascade (e.g., myocardial inflammation and oxidative stress), reduce myocardial injury, and improve functional independence. In the acute stage of ischemic cascade, the reduction in blood flow results in anaerobic metabolism and decreased molecular energy production. This, in turn, causes increased sodium influx and potassium efflux, disruption of ionic homeostasis and excitatory glutamate release, cellular edema, and secondary inflammation 34. Uninhibited glutamate release leads to mitochondrial dysfunction, free radical formation, and expansion of the infarct. Hypothermia only induced in ischemia can improve cardiomyocyte contractility and mitochondrial respiratory function in the model of cultured cardiomyocyte 51. Additionally, TH preserves mitochondrial integrity and viability of cardiomyocytes after ischemia-reperfusion injury 52. Putting these observations together, it appears that TH can alleviate the deteriorations of oxidative stress and inflammation via preserving mitochondrial integrity and viability of cardiomyocytes after heat stroke in rats.

**Figure 5:**
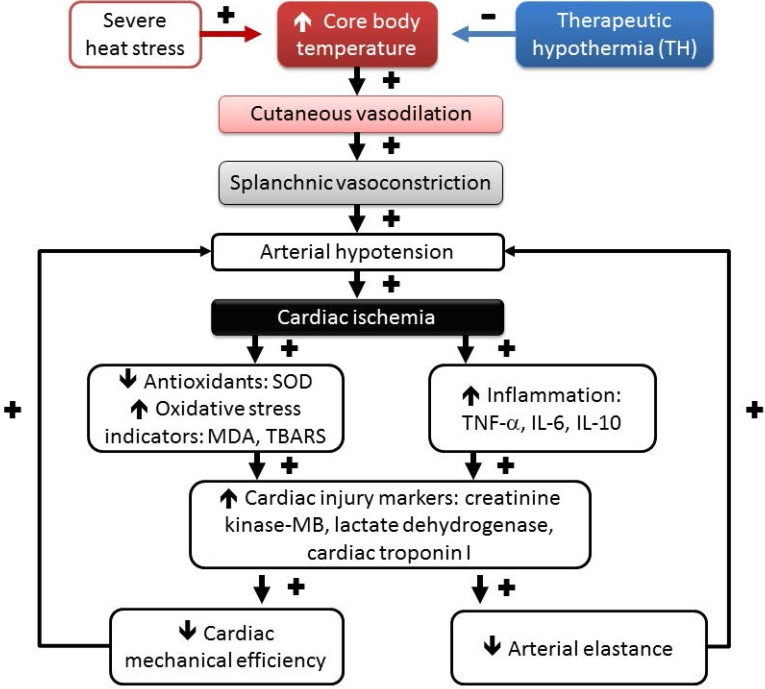
Temporal associations are driving the HS reactions, including hyperthermia, hypotension, splanchnic vasoconstriction, cardiac oxidative stress, cardiac inflammation, and lethality following heat stress, which can be affected by therapeutic hypothermia (TH) treatment. (+), aggravation, and (-), mitigation. Environmental heat stress increases cutaneous blood flow and metabolism and progressively decreases splanchnic blood flow (including heart, intestine, brain, and others). Decreasing mean arterial pressure by heat stress causes myocardial ischemia. Myocardial ischemia causes myocardial oxidative stress and inflammation and results in a reduction in both cardiac mechanical efficiency and arterial elastance, causing further hypotension. SOD, superoxide dismutase; MDA, malondialdehyde; TBARS, thiobarbituric acid reactive substances; TNF-α, tumor necrosis factor-alpha; IL-6, interleukin-6; IL-10, interleukin-10.

As depicted in Figure 5, environmental heat stress increases body core temperature, cutaneous blood flow, and metabolism and progressively decreases splanchnic blood flow 21, which results in arterial hypotension and myocardial ischemia. In heated rats, myocardial ischemia, inflammatory, and oxidative damage might cause cardiac cell death by apoptosis and autophagy 40, 53. Myocardial injury caused by severe heat ed rats, the stress might cause further arterial hypotension by decreasing both cardiac mechanical efficiency and arterial elastance and result in vital organ ischemia 19. Decreasing the whole-body temperature with TH (~32°C) significantly attenuates cardiac ischemic, inflammatory and oxidative damage, arterial hypotension (by decreasing cardiac mechanical efficiency and arterial elastance), and multiple organ ischemia and injury (Figure 5). Thus, we conclude that TH reverses the suppressed LV performance and subsequently maintains normal levels of arterial blood pressure in rats. Finally, it should be stressed that the effect of TH on the survival of HS rats may be related to its systemic, noncardiovascular effects (for instance, neurologic protection). The major limitation of the present study is that we focused mainly on cardiac protection.

## Conclusions

In summary, severe heat stress (43°C for 70 min) caused hyperthermia (~42°C), hypotension (~30 mmHg), and suppressed LV performance (due to myocardial inflammatory and oxidative injury) in heated rats. In addition, the percent survival was decreased from a value of 100% in the normothermic controls to a new value of 0% in the heated rats without TH. However, when applied during myocardial ischemia (due to hypotension), TH significantly reversed the myocardial injury and dysfunction, preserved an adequate blood supply to vital organs and subsequently improved survival in heated rats.

## Figures and Tables

**Table 1:** Hemodynamic parameters and indexes of systolic and diastolic function derived from left pressure-volume relationships in NC, HS rats without TH (HS+non-TH), and HS rats with TH (HS+TH).

Group of rats		NC rats			HS+non-TH			HS+TH	
Time points		0 min	90 min		0 min	90 min		0 min	90 min
**Parameters**									
HR, beats/min		344±6	349±5		351±7	53±4^*^		347±6	512±12^+^
ESP, mmHg		117±4	119±3		119±5	15±1		118±2	71±4^+^
EDP, mmHg		11±1	10±1		12±1	5±1^*^		10±1	8±1^+^
SV, μl		122±5	125±4		124±3	52±4^*^		123±2	97±5^+^
ESV, μl		298±6	303±5		302±4	418±9^*^		299±5	305±6^+^
EDV, μl		409±5	416±7		401±6	459±5^*^		404±5	402±6^+^
CO, μl/min		44,662±32	44,594±37		44,686±39	2,982±22^*^		44,573±	45,740±35^+^
**Systolic indexes**									
EF, %		0.65±0.03	0.69±0.04		0.66±0.04	0.29±0.02^*^		0.64±0.04	0.65±0.03^+^
Maximal dp/dt, mmHg/s		9,982±8	9,990±12		9,988±18	1,492±37^*^		9,991±12	6,176±98^+^
Pmax, mmHg		144±5	139±4		139±6	37±3^*^		137±5	93±5^+^
Vmax, μl/s		408±7	416±7		414±6	514±7^*^		409±6	428±5^+^
SW, mmHg/μl		13,395±81	13,406±83		13,416±74	1,258±64^*^		13,395±77	9,121±107^+^
**Diastolic indexes**									
Minimum do/dt, mmHg		-5,600±18	-5,692±25		-5,688±22	-159±15^*^		-5,697±33	-4,416±125^+^
Pmin, mmHg		293±6	288±9		301±8	404±9^*^		288±7	313±7^+^
**Relaxation factor (γ)**									
ms		12±0	12±0		12±0	72±1^*^		12±0	39±1^+^
**Aortic vascular index**									
Ea, mmHg/μl		0.99±0.02	10.1±0.3		0.98±0.02	0.31±0.02^*^		0.97±0.02	0.99±0.02^+^

Values are expressed as means±SEM; n=10 for each group. HR, heart rate; ESP, end-systolic pressure; EDP, end-diastolic pressure; SV, stroke volume; ESV, LV end-systolic pressure; SV, stroke volume; ESV, LV end-systolic volume; Pmax, maximum LV pressure; Vmax, maximum dv/dt; Pmin, minimum LV pressure; γ, relaxation factor (Tau Glantz or time constant of ventricular relaxation); Ea, arterial elastance; and Vmin, minimum dp/dt. *P<0.05 *vs* NC. +*P*<0.05 *vs* HS+non-TH.

**Table 2:** Levels of myocardial injury markers, myocardial antioxidant and oxidative stress markers, and myocardial inflammatory cytokines in NC, HS rats without TH (HS+non-TH), and HS rats with TH (HS+TH).

Groups of rats		(NC)	(HS+non-TH)	(HS+TH)
**Parameters**				
**Myocardial injury markers**				
Creatine kinase-MB (U/L)		24±4	86±9^*^	37±6^+^
Lactate dehydrogenase (U/L)		51±8	155±12^*^	76±9^+^
Cardiac troponin I (ng/ml)		0.57±0.08	3.08±0.72^*^	1.06±0.11^+^
**Myocardial antioxidant and markers oxidative stress markers**				
MDA (nmol/mg)		1.06±0.19	6.52±1.02^*^	2.68±0.63^+^
TBARS (nmol/g)		2±1	29±4^*^	12±3^+^
SOD (unit/mg)		17±3	6±2^*^	13±3^+^
Catalase (nM of H_2_O_2_- consumption/min/mg)		9±2	5±2^*^	10±3^+^
GSH (mmol/mg)		2.53±0.24	0.64±0.32^*^	2.71±0.26^+^
**Inflammatory cytokines**				
TNF-α (pg/ml)		1000±380	3200±310^*^	1500±320^+^
IL-6 (pg/ml)		102±24	668±85^*^	108±35^+^
IL-10 (pg/ml)		3008±660	992±227^*^	2955±441^+^

Values are expressed as the means±SD; n=10 for each group. MDA, malondialdehyde; TBARS, thiobarbituric acid-reactive substances; SOD, superoxide dismutase; TNF-α, tumor necrosis factor-alpha; IL-6, interleukin-6; and IL-10, interleukin-10. ^*^P<0.05 *vs* NC. ^+^*P*<0.05 *vs* HS+TH.

## Abbreviations

TH: Therapeutic hypothermia
HR: Heart rate
ESP: Left ventricular (LV) end-systolic pressure
EDP: LV end-diastolic pressure
SV: Stroke volume
ESV: LV end-systolic pressure
EDV: LV end-diastolic volume
CO: Cardiac output
EF: Ejection fraction
Pmax: Maximal LV pressure
Vmax: Maximal LV volume
SW: Stroke work
Pmin: Minimum LV pressure
Tau: Glantz time constant of ventricular relaxation
MDA: Malondialdehyde
TBARS: Thiobarbituric acid-reactive substances
SOD: Superoxide dismutase
DTNB: Dithionitrobenzoic acid
GSH: Reduced glutathione
TNF-α: Tumor necrosis factor-α
IL-1β: Interleukin-1β
